# Crystal structures of 1-benzene­sulfon­yl-2-methyl-3-(4-nitro­benzoyl)-2,3-di­hydro-1*H*-indole and 1-benzene­sulfon­yl-2-methyl-3-[(thio­phen-2-yl)carbon­yl]-2,3-di­hydro-1*H*-indole

**DOI:** 10.1107/S2056989017012804

**Published:** 2017-09-29

**Authors:** G. Dhanalakshmi, Velu Saravanan, Arasambattu K. Mohanakrishnan, S. Aravindhan

**Affiliations:** a Department of Physics, Misrimal Navajee Munoth Jain Engineering College, Chennai, 600 097, India; b Department of Physics, Presidency College (Autonomous), Chennai 600 005, India; c Department of Organic Chemistry, University of Madras, Guindy Campus, Chennai, 600 025, India

**Keywords:** crystal structure, indole, nitro­phen­yl, thio­phen:(phen­yl)methanone, inter- mol­ecular and intra mol­ecular hydrogen bonds

## Abstract

In the title indole derivatives, C_22_H_16_N_2_O_5_S, (I) and C_20_H_15_NO_3_S_2_, (II), the sulfonyl-bound phenyl rings are almost orthogonal to the indole ring system, subtending dihedral angles of 88.33 (10) and 87.58 (16)°, respectively. In both compounds, the sulfonyl S atom has a distorted tetra­hedral geometry [O—S—O = 119.98 (9) and N—S—C = 104.01 (8)° for compound (I) and O—S—O = 120.08 (18) and N—S—C = 104.91 (14)° for compound (II)] and the sum of the bond angles at N indicates *sp* ^2^ hybridization. The mol­ecules of both (I) and (II) feature intra­molecular C—H⋯O hydrogen bonds that generate *S*(6) ring motifs with the sulfone O atom. In the crystals, mol­ecules of (I) are linked by C—H—O hydrogen bonds, forming *R* ^4^_4_(18) ring motifs while mol­ecules of (II) are linked by C—H—O and C—H—S hydrogen bonds, forming *R* ^2^_2_(12) ring motifs. Compound (II) was refined as an inversion twin.

## Abstract 2

In the title indole derivatives, the sulfonyl-bound phenyl rings are almost orthogonal to the indole ring system, subtending dihedral angles of 88.33 (10) and 87.58 (16)°, respectively. The mol­ecules of both (I) and (II) feature intra­molecular C—H⋯O hydrogen bonds that generate *S*(6) ring motifs with the sulfone O atom. In the crystals, mol­ecules of (I) are linked by C—H—O hydrogen bonds, forming 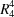
(18) ring motifs while mol­ecules of (II) are linked by C—H—O and C—H—S hydrogen bonds, forming 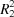
(12) ring motifs.

## Chemical context   

Indole is the parent compound of a large number of important compounds in nature with significant biological activity (Kaushik *et al.*, 2013 ▸). Indole derivatives are known to exhibit anti-bacterial, anti-fungal (Singh *et al.*, 2000 ▸), anti-tumour (Andreani *et al.*, 2001 ▸), anti­depressant (Grinev *et al.*, 1984 ▸), anti-inflammatory (Rodriguez *et al.*, 1985 ▸) and physiological (Porter *et al.*, 1977 ▸; Sundberg, 1996 ▸) properties. They are used as bioactive drugs (Stevenson *et al.*, 2000 ▸) and have also been proven to display high aldose reductase inhibitory (Rajeswaran *et al.*, 1999 ▸) and anti­microbial activities (Amal Raj *et al.*, 2003 ▸). Indole deriv­atives containing a phenyl­sulfonyl group exhibit insecticidal, germicidal and fungicidal activity (Wolf, 1999 ▸). Against this background, the crystal structure determination of the title compounds was carried out to study their structural aspects and the results are presented here.

## Structural commentary   

**Figure 1:**
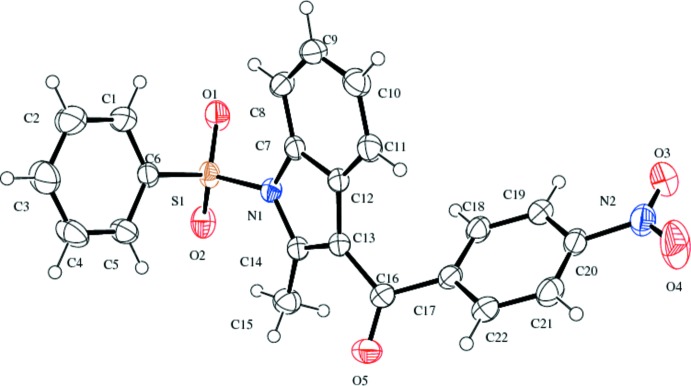
The mol­ecular structure of compound (I) with the atom labelling. Displacement ellipsoids are drawn at the 40% probability level.

The mol­ecular structure of compound (I) is shown in Fig. 1 ▸. The geometric parameters are in close agreement with those of similar structures. (Umadevi *et al.*, 2015*a* ▸,*b* ▸). The sulfonyl-bound phenyl ring (C1–C6) is almost orthogonal to the indole ring system (N1/C7-C14) making a dihedral angle of 88.43 (10)°·The nitro­phenyl ring (C17–C22) forms a dihedral angle of 61.00 (8)° with the indole ring system. The dihedral angle between the phenyl rings is 77.97 (11)°. The C16—C13—C14—N1 torsion angle is 174.58 (16)°. The sum of the bond angles at N1 (357.7°) indicates *sp* ^2^ hybridization (Beddoes *et al.*, 1986 ▸).
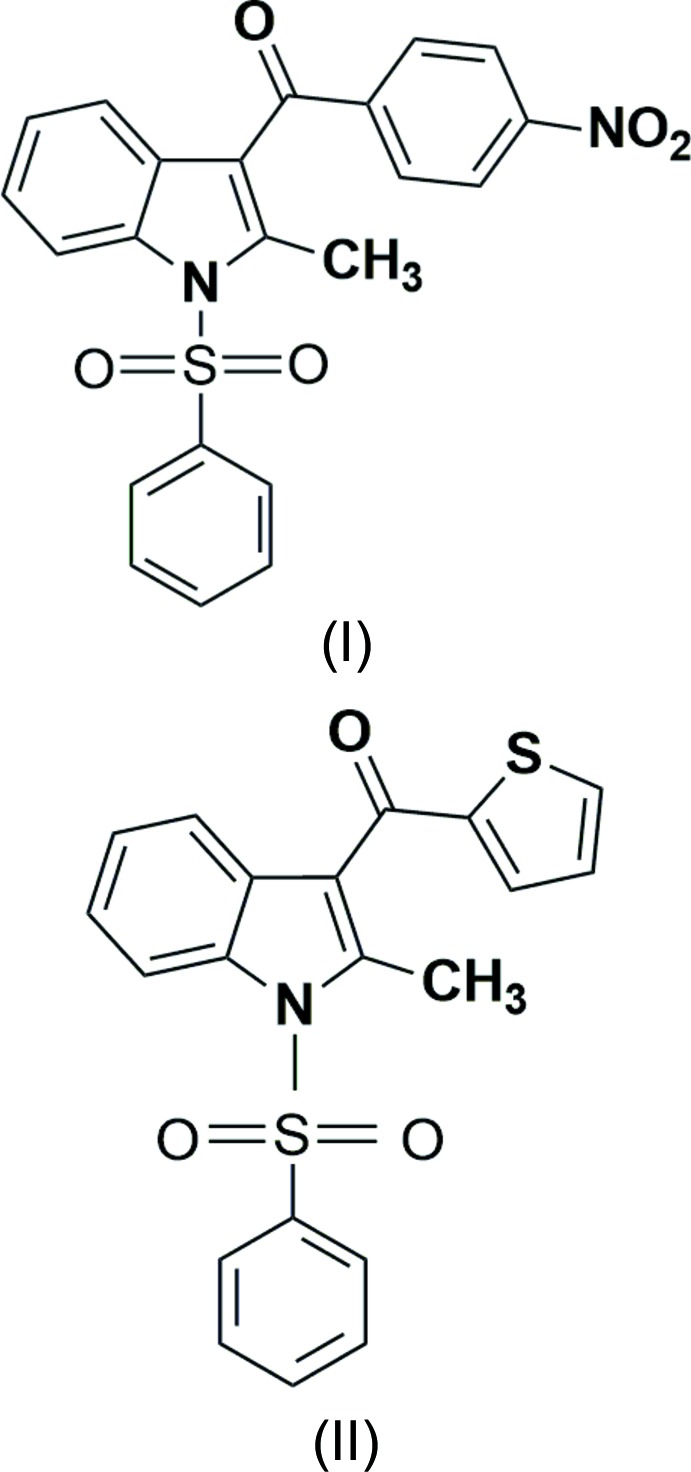


**Figure 2:**
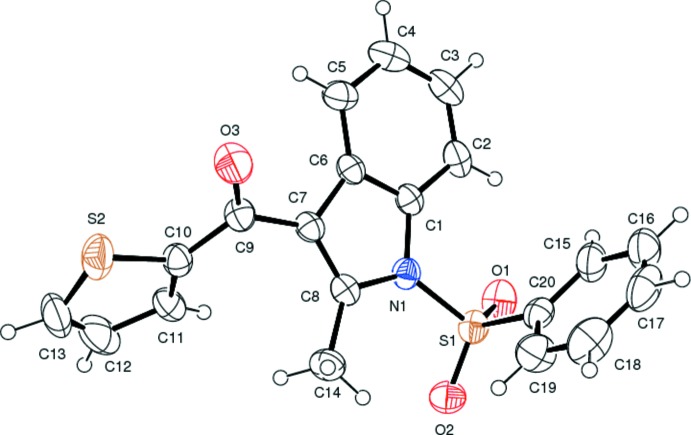
The mol­ecular structure of compound (II) with the atom labelling. Displacement ellipsoids are drawn at the 40% probability level.

The mol­ecular structure of compound (II) is shown in Fig. 2 ▸. The geometric parameters of (II) are in close agreement with those of similar structures (KamalaKumar *et al.*, 2011 ▸). The sulfonyl-bound phenyl ring (C15–C20) is almost orthogonal to the indole ring system (N1/C1–C8), making a dihedral angle of 87.58 (16)°. The dihedral angle between the indole moiety (N1/C1–C8) and the thio­phene ring (S2 /C10–C13) is 56.05 (19)° while that between the thio­phene and phenyl rings is 54.0 (2)°. The C9—C7—C8—N1 torsion angles is 178.5 (3)°. The sum of the bond angles around N1 is 358.4°, indicating *sp* ^2^ hybridization.

In both compounds, the indole moiety is essentially planar with a maximum deviation of 0.021 (2) Å for both atom C10 in compound (I) and atom C8 in compound (II). In both compounds, the variation in endocyclic angles [119.05 (16)° at C12 and 122.17 (17)° at C7 for compound (I) and 119.7 (3)° at C6 and 121.5 (3)° at C1 for compound (II)] of the benzene ring of the indole ring system are due to the fusion of the five- and six -membered rings and the strain is taken up by the angular distortion rather than by bond-length distortion (Allen *et al.*, 1987 ▸).

Atom S1 has a distorted tetra­hedral configuration with angles O1—S1—O2 = 119.98 (9) and N1—S1—C6 = 104.01 (8)° for compound (I) and O1—S1—O2 = 120.08 (18) and N1—S1—C20 = 104.91 (14)° for compound (II), differing from the ideal tetra­hedral values attributing to the Thorpe–Ingold effect (Bassindale,1984 ▸). As a result of the electron-withdrawing character of the phenyl­sulfonyl group, in both compounds the N—C bond lengths [N1—C7 = 1.418 (2) and N1—C14 = 1.412 (2) Å for compound (I) and N1—C1 = 1.413 (4) and N1—C8 = 1.421 (4) Å for compound (II) are longer than the mean value of 1.355 (14) Å (Allen *et al.*, 1987 ▸). In both compounds, the mol­ecules are stabilized by intra­molecular C—H⋯O hydrogen bonds (Tables 1 ▸ and 2 ▸), which generate *S*(6) ring motifs with the sulfone oxygen atoms.

## Supra­molecular features   

**Figure 3:**
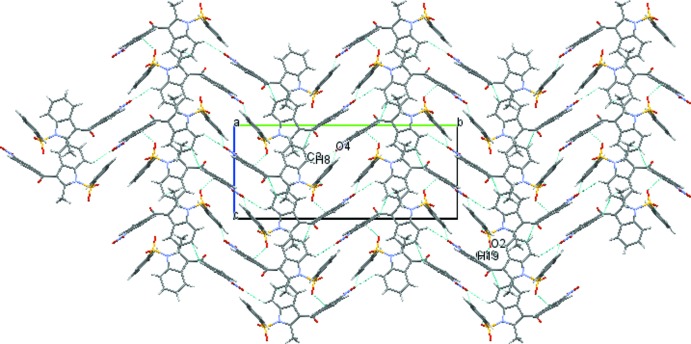
The crystal packing of compound (I) viewed along the *a* axis, showing the inter­molecular C8—H8—O4 and C19—H19—O2 hydrogen bonds as dashed lines. Symmetry codes are as in Table 1 ▸.

**Figure 4:**
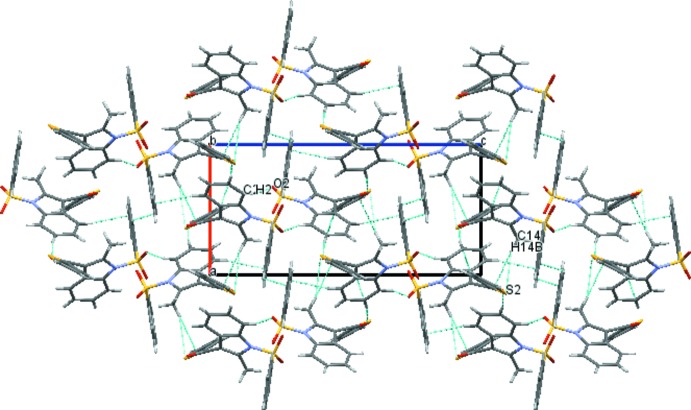
The crystal packing of compound (II) viewed along the *b* axis, showing the inter­molecular C2—H2—O2 and C14—H14*B*—S2 hydrogen bonds as dashed lines. Symmetry codes are as in Table 2 ▸.

In the crystal of (I), the mol­ecules are linked *via* C8—H8⋯O4^i^ and C19—H19⋯O2^ii^ hydrogen bonds (Fig. 3 ▸), forming 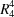
(18) motifs (two-dimensional network). In the crystal of (II), the mol­ecules are linked *via* C2—H2⋯O2^i^ and C14—H14*B*⋯S2^ii^ hydrogen bonds (Fig. 4 ▸), forming 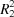
(12) motifs (two-dimensional network). No significant π–π or C—H⋯π inter­actions are observed in either compound.

## Database survey   

A search of the Cambridge Structural Database (Groom *et al.*, 2016 ▸) yielded 67 hits for the 1-phenyl­sulfonyl-1*H*-indole moiety and 49 hits for 2-methyl-1-phenyl­sulfonyl-1*H*-indole-3-yl). The compound (2-methyl-1-phen­ylsulfonyl-1*H*-indol-3-yl)(phen­yl)methanone (LOSMEN; Umadevi *et al.*, 2015*a* ▸), which crystallizes in the *P*2_1_2_1_2_1_ space group, is the closest analogue of compound (I). The compound (1-phen­ylsulfonyl-1*H*-indol-2-yl)(thio­phen-2-yl)methanone (ULINEJ; KamalaKumar *et al.*, 2011 ▸), which crystallizes in space group *P*1, is the closest analogue of compound (II). The packing of compounds (I) and (II) feature C—H⋯O and C—H⋯S inter­actions, but the related structures exhibit C—H⋯O and C—H⋯π inter­actions. In the latter structures, the sulfonyl-bound phenyl ring is almost orthogonal to the indole ring system, making dihedral angles of 84.89 (7) and 54.91 (11)°, respectively, comparable with those observed in the title compounds.

## Synthesis and crystallization   

**Compound (I)**

To a solution of 4-nitro­benzoyl chloride (2.06 g, 11.07 mmol) in dry DCM (15 ml) at 273 K, SnCl_4_ (2.06 g, 11.07 mmol) was added slowly (5 min). To this, a solution of 1-phenyl­sulfonyl-2-methyl­indole (2 g, 7.38 mmol) in dry DCM (10 ml) was added (5 min) and allowed to stir at room temperature for 48 h. After completion of the reaction (monitored by TLC), it was poured into ice–water (50 ml) containing conc. HCl (10 ml). The organic layer was separated and the aqueous layer was extracted with DCM (2 × 20 ml). The combined organic layer was washed with water (3 × 25 ml) and dried (Na_2_SO_4_). The subsequent purification of the crude product either by washing with MeOH or column chromatography (silica gel, hexa­ne:ethyl acetate 8:2) furnished the first title compound as a colourless solid (1.92 g, 62%); m.p. 435–437 K.

**Compound (II)**

To a solution of thio­phene-2-carbonyl chloride (1.63 g, 11.07 mmol) and SnCl_4_ (2.88 g, 11.07 mmol) in dry DCM (20 ml) at 273 K, a solution of 1-phenyl­sulfonyl-2-methyl­indole (2 g, 7.38 mmol) in dry DCM (10 ml) was added slowly (5 min). Then, it was stirred at room temperature for 30 min. After completion of the reaction (monitored by TLC), it was poured into ice–water (50 ml) containing conc. HCl (10 ml). The organic layer was separated and the aqueous layer was extracted with DCM (2 × 20 ml). The combined organic extract was washed with water (3 × 25 ml) and dried (Na_2_SO_4_). Evaporation of the solvent followed by trituration of the crude product with MeOH (5 ml) gave the second title compound as a colourless solid (2.19 g, 78%); m.p. 379–381 K.

## Refinement   

Crystal data, data collection and structure refinement details are summarized in Table 3 ▸. H atoms were localized from the difference electron-density maps and refined as riding atoms with C—H = 0.93 or 0.97 Å with *U*_iso_(H) = 1.5*U*_eq_(C) for methyl H atoms and 1.2*U*_eq_(C) for other H atoms. Compound (II) was refined as an inversion twin (BASF 0.03).

## Supplementary Material

Crystal structure: contains datablock(s) I, II. DOI: 10.1107/S2056989017012804/ff2151sup1.cif

Structure factors: contains datablock(s) I. DOI: 10.1107/S2056989017012804/ff2151Isup2.hkl

Supporting information file. DOI: 10.1107/S2056989017012804/ff2151Isup4.cml

Structure factors: contains datablock(s) II. DOI: 10.1107/S2056989017012804/ff2151IIsup3.hkl

Supporting information file. DOI: 10.1107/S2056989017012804/ff2151IIsup5.cml

CCDC references: 1561045, 1561044

Additional supporting information:  crystallographic information; 3D view; checkCIF report

## Figures and Tables

**Table 1:** Hydrogen-bond geometry (Å, °) for (I)

*D*—H⋯*A*	*D*—H	H⋯*A*	*D*⋯*A*	*D*—H⋯*A*
C8—H8⋯O1	0.93	2.40	2.977 (3)	120
C8—H8⋯O4^i^	0.93	2.60	3.286 (2)	131
C15—H15*A*⋯O2	0.96	2.03	2.824 (3)	139
C19—H19⋯O2^ii^	0.93	2.64	3.388 (2)	138

Symmetry codes: (i) 
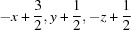
; (ii) 
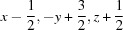
.

**Table 2:** Hydrogen-bond geometry (Å, °) for (II)

*D*—H⋯*A*	*D*—H	H⋯*A*	*D*⋯*A*	*D*—H⋯*A*
C2—H2⋯O1	0.93	2.39	2.954 (5)	119
C2—H2⋯O2^i^	0.93	2.65	3.394 (4)	138
C14—H14*A*⋯O2	0.96	2.00	2.806 (4)	140
C14—H14*B*⋯S2^ii^	0.96	2.93	3.822 (4)	156

Symmetry codes: (i) 
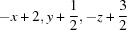
; (ii) 
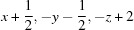
.

**Table 3:** Experimental details

	(I)	(II)
Crystal data		
Chemical formula	C_22_H_16_N_2_O_5_S	C_20_H_15_NO_3_S_2_
*M*_r_	420.43	381.45
Crystal system, space group	Monoclinic, *P*2_1_/*n*	Orthorhombic, *P*2_1_2_1_2_1_
Temperature (K)	296	293
*a*, *b*, *c* (Å)	8.1358 (2), 23.8364 (7), 10.5983 (3)	8.9300 (2), 10.8141 (3), 18.6398 (5)
α, β, γ (°)	90, 110.210 (1), 90	90, 90, 90
*V* (Å^3^)	1928.77 (9)	1800.04 (8)
*Z*	4	4
Radiation type	Cu *K*α	Cu *K*α
μ (mm^−1^)	1.83	2.85
Crystal size (mm)	0.20 × 0.15 × 0.15	0.25 × 0.20 × 0.15

Data collection		
Diffractometer	Bruker Kappa APEX3 CMOS	Bruker Kappa APEX3 CMOS
Absorption correction	Multi-scan (*SADABS*; Bruker, 2016 ▸)	Multi-scan (*SADABS*; Bruker, 2016 ▸)
*T*_min_, *T*_max_	0.657, 0.754	0.599, 0.746
No. of measured, independent and observed [*I* > 2σ(*I*)] reflections	30940, 3780, 3379	25415, 3538, 3314
*R*_int_	0.042	0.043
(sin θ/λ)_max_ (Å^−1^)	0.619	0.618

Refinement		
*R*[*F* ^2^ > 2σ(*F* ^2^)], *wR*(*F* ^2^), *S*	0.042, 0.118, 1.07	0.039, 0.106, 1.06
No. of reflections	3780	3538
No. of parameters	271	236
H-atom treatment	H-atom parameters constrained	H-atom parameters constrained
Δρ_max_, Δρ_min_ (e Å^−3^)	0.45, −0.39	0.24, −0.38
Absolute structure	–	Refined as an inversion twin
Absolute structure parameter	–	0.03 (3)

Computer programs: *APEX3*, *SAINT* and *XPREP* (Bruker, 2016 ▸), *SHELXT2014/7* (Sheldrick, 2015*a* ▸), *SHELXL2014/7* (Sheldrick, 2015*b* ▸), *ORTEP-3 for Windows* (Farrugia, 2012 ▸) and *Mercury* (Macrae *et al.*, 2008 ▸).

## supplementary crystallographic information

### 1-Benzenesulfonyl-2-methyl-3-(4-nitrobenzoyl)-2,3-dihydro-1H-indole (I) Crystal data

**Table:**

C_22_H_16_N_2_O_5_S	*F*(000) = 872
*M**_r_* = 420.43	*D*_x_ = 1.448 Mg m^−^^3^
Monoclinic, *P*2_1_/*n*	Cu *K*α radiation, λ = 1.54178 Å
*a* = 8.1358 (2) Å	Cell parameters from 9895 reflections
*b* = 23.8364 (7) Å	θ = 3.7–72.4°
*c* = 10.5983 (3) Å	µ = 1.83 mm^−^^1^
β = 110.210 (1)°	*T* = 296 K
*V* = 1928.77 (9) Å^3^	Block, colourless
*Z* = 4	0.20 × 0.15 × 0.15 mm

### 1-Benzenesulfonyl-2-methyl-3-(4-nitrobenzoyl)-2,3-dihydro-1H-indole (I) Data collection

**Table:**

Bruker Kappa APEX3 CMOS diffractometer	3780 independent reflections
Radiation source: micro-focus sealed tube	3379 reflections with *I* > 2σ(*I*)
Graphite monochromator	*R*_int_ = 0.042
ω and φ scan	θ_max_ = 72.5°, θ_min_ = 3.7°
Absorption correction: multi-scan (SADABS; Bruker, 2016)	*h* = −10→10
*T*_min_ = 0.657, *T*_max_ = 0.754	*k* = −29→29
30940 measured reflections	*l* = −13→12

### 1-Benzenesulfonyl-2-methyl-3-(4-nitrobenzoyl)-2,3-dihydro-1H-indole (I) Refinement

**Table:**

Refinement on *F*^2^	0 restraints
Least-squares matrix: full	Hydrogen site location: inferred from neighbouring sites
*R*[*F*^2^ > 2σ(*F*^2^)] = 0.042	H-atom parameters constrained
*wR*(*F*^2^) = 0.118	*w* = 1/[σ^2^(*F*_o_^2^) + (0.0653*P*)^2^ + 0.7114*P*] where *P* = (*F*_o_^2^ + 2*F*_c_^2^)/3
*S* = 1.07	(Δ/σ)_max_ < 0.001
3780 reflections	Δρ_max_ = 0.45 e Å^−^^3^
271 parameters	Δρ_min_ = −0.39 e Å^−^^3^

### 1-Benzenesulfonyl-2-methyl-3-(4-nitrobenzoyl)-2,3-dihydro-1H-indole (I) Special details

**Table:**

Geometry. All esds (except the esd in the dihedral angle between two l.s. planes) are estimated using the full covariance matrix. The cell esds are taken into account individually in the estimation of esds in distances, angles and torsion angles; correlations between esds in cell parameters are only used when they are defined by crystal symmetry. An approximate (isotropic) treatment of cell esds is used for estimating esds involving l.s. planes.

### 1-Benzenesulfonyl-2-methyl-3-(4-nitrobenzoyl)-2,3-dihydro-1H-indole (I) Fractional atomic coordinates and isotropic or equivalent isotropic displacement parameters (Å^2^)

**Table:**

	*x*	*y*	*z*	*U*_iso_*/*U*_eq_	
C1	1.0703 (3)	0.92527 (10)	0.0217 (3)	0.0560 (6)	
H1	0.9759	0.9376	0.0448	0.067*	
C2	1.2366 (3)	0.94573 (12)	0.0873 (3)	0.0730 (8)	
H2	1.2548	0.9720	0.1557	0.088*	
C3	1.3757 (3)	0.92775 (12)	0.0526 (3)	0.0662 (7)	
H3	1.4869	0.9423	0.0962	0.079*	
C4	1.3504 (3)	0.88822 (12)	−0.0466 (2)	0.0587 (6)	
H4	1.4452	0.8756	−0.0686	0.070*	
C5	1.1860 (3)	0.86712 (9)	−0.1135 (2)	0.0446 (4)	
H5	1.1687	0.8405	−0.1810	0.054*	
C6	1.0471 (2)	0.88612 (7)	−0.07867 (18)	0.0351 (4)	
C7	0.8417 (2)	0.79012 (7)	0.03534 (17)	0.0337 (4)	
C8	0.8116 (3)	0.82909 (8)	0.1227 (2)	0.0419 (4)	
H8	0.7817	0.8660	0.0962	0.050*	
C9	0.8278 (3)	0.81092 (9)	0.2498 (2)	0.0464 (5)	
H9	0.8063	0.8359	0.3097	0.056*	
C10	0.8757 (3)	0.75604 (9)	0.2906 (2)	0.0468 (5)	
H10	0.8893	0.7452	0.3780	0.056*	
C11	0.9033 (2)	0.71729 (8)	0.20276 (18)	0.0406 (4)	
H11	0.9344	0.6806	0.2302	0.049*	
C12	0.8837 (2)	0.73419 (7)	0.07227 (17)	0.0333 (4)	
C13	0.9047 (2)	0.70602 (7)	−0.04222 (18)	0.0347 (4)	
C14	0.8782 (2)	0.74412 (7)	−0.14365 (18)	0.0352 (4)	
C15	0.8744 (3)	0.73343 (9)	−0.2837 (2)	0.0481 (5)	
H15A	0.8532	0.7680	−0.3331	0.072*	
H15B	0.9849	0.7182	−0.2806	0.072*	
H15C	0.7827	0.7072	−0.3272	0.072*	
C16	0.9371 (3)	0.64522 (7)	−0.05201 (19)	0.0387 (4)	
C17	0.8454 (2)	0.60596 (7)	0.01248 (18)	0.0365 (4)	
C18	0.6765 (3)	0.61765 (7)	0.00916 (19)	0.0406 (4)	
H18	0.6205	0.6501	−0.0330	0.049*	
C19	0.5910 (3)	0.58154 (8)	0.06783 (19)	0.0419 (4)	
H19	0.4772	0.5887	0.0644	0.050*	
C20	0.6803 (3)	0.53438 (7)	0.13178 (18)	0.0402 (4)	
C21	0.8460 (3)	0.52090 (8)	0.1341 (2)	0.0460 (5)	
H21	0.9009	0.4882	0.1754	0.055*	
C22	0.9286 (3)	0.55716 (8)	0.07365 (19)	0.0424 (4)	
H22	1.0404	0.5489	0.0739	0.051*	
N1	0.83594 (19)	0.79620 (6)	−0.09917 (15)	0.0355 (3)	
N2	0.5943 (3)	0.49781 (7)	0.20229 (18)	0.0523 (4)	
O1	0.71132 (17)	0.89267 (6)	−0.13460 (16)	0.0512 (4)	
O2	0.81830 (19)	0.85052 (6)	−0.30381 (14)	0.0496 (4)	
O3	0.4452 (3)	0.50868 (8)	0.19518 (19)	0.0681 (5)	
O4	0.6773 (3)	0.45810 (9)	0.2654 (2)	0.0889 (7)	
O5	1.0299 (2)	0.62677 (6)	−0.11082 (17)	0.0579 (4)	
S1	0.83727 (5)	0.85977 (2)	−0.16730 (5)	0.03710 (15)	

### 1-Benzenesulfonyl-2-methyl-3-(4-nitrobenzoyl)-2,3-dihydro-1H-indole (I) Atomic displacement parameters (Å^2^)

**Table:**

	*U*^11^	*U*^22^	*U*^33^	*U*^12^	*U*^13^	*U*^23^
C1	0.0467 (11)	0.0513 (12)	0.0793 (15)	−0.0074 (9)	0.0336 (11)	−0.0216 (11)
C2	0.0570 (14)	0.0802 (17)	0.0873 (18)	−0.0211 (13)	0.0319 (13)	−0.0427 (15)
C3	0.0393 (11)	0.0891 (19)	0.0686 (15)	−0.0184 (11)	0.0167 (10)	−0.0237 (13)
C4	0.0322 (10)	0.0888 (17)	0.0585 (13)	−0.0028 (10)	0.0199 (9)	−0.0153 (12)
C5	0.0365 (10)	0.0551 (11)	0.0444 (10)	0.0004 (8)	0.0167 (8)	−0.0068 (8)
C6	0.0323 (8)	0.0303 (8)	0.0453 (9)	−0.0006 (7)	0.0166 (7)	0.0042 (7)
C7	0.0295 (8)	0.0317 (8)	0.0423 (9)	−0.0037 (6)	0.0154 (7)	0.0008 (7)
C8	0.0414 (10)	0.0321 (9)	0.0542 (11)	−0.0022 (7)	0.0189 (8)	−0.0067 (8)
C9	0.0445 (10)	0.0476 (11)	0.0519 (11)	−0.0070 (8)	0.0228 (9)	−0.0153 (9)
C10	0.0484 (11)	0.0541 (12)	0.0405 (10)	−0.0078 (9)	0.0189 (8)	−0.0039 (8)
C11	0.0427 (10)	0.0380 (9)	0.0431 (10)	−0.0019 (8)	0.0175 (8)	0.0037 (7)
C12	0.0313 (8)	0.0298 (8)	0.0416 (9)	−0.0031 (6)	0.0161 (7)	−0.0007 (7)
C13	0.0360 (9)	0.0297 (8)	0.0425 (9)	−0.0019 (7)	0.0190 (7)	0.0007 (7)
C14	0.0344 (9)	0.0312 (8)	0.0432 (9)	−0.0021 (7)	0.0173 (7)	0.0004 (7)
C15	0.0574 (12)	0.0472 (11)	0.0438 (10)	0.0036 (9)	0.0227 (9)	0.0021 (8)
C16	0.0436 (10)	0.0318 (9)	0.0438 (10)	0.0023 (7)	0.0191 (8)	0.0000 (7)
C17	0.0441 (10)	0.0280 (8)	0.0385 (9)	−0.0001 (7)	0.0159 (7)	−0.0022 (7)
C18	0.0443 (10)	0.0284 (8)	0.0488 (10)	0.0026 (7)	0.0158 (8)	0.0038 (7)
C19	0.0449 (10)	0.0340 (9)	0.0493 (10)	−0.0017 (8)	0.0194 (8)	−0.0044 (7)
C20	0.0552 (11)	0.0307 (9)	0.0366 (9)	−0.0085 (8)	0.0182 (8)	−0.0024 (7)
C21	0.0581 (12)	0.0315 (9)	0.0473 (10)	0.0061 (8)	0.0169 (9)	0.0073 (7)
C22	0.0475 (10)	0.0325 (9)	0.0491 (10)	0.0057 (8)	0.0192 (8)	0.0002 (7)
N1	0.0379 (8)	0.0283 (7)	0.0434 (8)	−0.0017 (6)	0.0178 (6)	0.0021 (6)
N2	0.0705 (12)	0.0409 (9)	0.0492 (9)	−0.0120 (8)	0.0254 (9)	0.0000 (7)
O1	0.0350 (7)	0.0394 (7)	0.0815 (10)	0.0079 (6)	0.0229 (7)	0.0124 (7)
O2	0.0500 (8)	0.0494 (8)	0.0436 (7)	−0.0066 (6)	0.0086 (6)	0.0116 (6)
O3	0.0760 (12)	0.0623 (10)	0.0812 (12)	−0.0144 (9)	0.0462 (10)	0.0014 (9)
O4	0.0938 (15)	0.0723 (13)	0.1039 (15)	0.0029 (11)	0.0386 (12)	0.0502 (12)
O5	0.0761 (11)	0.0427 (8)	0.0738 (10)	0.0102 (7)	0.0499 (9)	0.0026 (7)
S1	0.0300 (2)	0.0314 (2)	0.0490 (3)	0.00052 (15)	0.01240 (18)	0.00904 (17)

### 1-Benzenesulfonyl-2-methyl-3-(4-nitrobenzoyl)-2,3-dihydro-1H-indole (I) Geometric parameters (Å, º)

**Table:**

C1—C6	1.378 (3)	C13—C16	1.483 (2)
C1—C2	1.379 (3)	C14—N1	1.412 (2)
C1—H1	0.9300	C14—C15	1.495 (3)
C2—C3	1.374 (4)	C15—H15A	0.9600
C2—H2	0.9300	C15—H15B	0.9600
C3—C4	1.373 (3)	C15—H15C	0.9600
C3—H3	0.9300	C16—O5	1.216 (2)
C4—C5	1.374 (3)	C16—C17	1.501 (2)
C4—H4	0.9300	C17—C22	1.389 (3)
C5—C6	1.381 (3)	C17—C18	1.391 (3)
C5—H5	0.9300	C18—C19	1.382 (3)
C6—S1	1.7561 (18)	C18—H18	0.9300
C7—C8	1.391 (2)	C19—C20	1.382 (3)
C7—C12	1.398 (2)	C19—H19	0.9300
C7—N1	1.418 (2)	C20—C21	1.378 (3)
C8—C9	1.378 (3)	C20—N2	1.473 (2)
C8—H8	0.9300	C21—C22	1.381 (3)
C9—C10	1.390 (3)	C21—H21	0.9300
C9—H9	0.9300	C22—H22	0.9300
C10—C11	1.384 (3)	N1—S1	1.6801 (14)
C10—H10	0.9300	N2—O3	1.217 (3)
C11—C12	1.396 (2)	N2—O4	1.219 (3)
C11—H11	0.9300	O1—S1	1.4250 (14)
C12—C13	1.447 (2)	O2—S1	1.4183 (15)
C13—C14	1.366 (2)		
			
C6—C1—C2	118.5 (2)	N1—C14—C15	123.86 (16)
C6—C1—H1	120.8	C14—C15—H15A	109.5
C2—C1—H1	120.8	C14—C15—H15B	109.5
C3—C2—C1	120.7 (2)	H15A—C15—H15B	109.5
C3—C2—H2	119.7	C14—C15—H15C	109.5
C1—C2—H2	119.7	H15A—C15—H15C	109.5
C4—C3—C2	120.0 (2)	H15B—C15—H15C	109.5
C4—C3—H3	120.0	O5—C16—C13	123.12 (17)
C2—C3—H3	120.0	O5—C16—C17	120.16 (16)
C3—C4—C5	120.5 (2)	C13—C16—C17	116.71 (15)
C3—C4—H4	119.7	C22—C17—C18	119.84 (17)
C5—C4—H4	119.7	C22—C17—C16	119.77 (17)
C4—C5—C6	118.84 (19)	C18—C17—C16	120.38 (16)
C4—C5—H5	120.6	C19—C18—C17	120.68 (17)
C6—C5—H5	120.6	C19—C18—H18	119.7
C1—C6—C5	121.48 (18)	C17—C18—H18	119.7
C1—C6—S1	120.22 (14)	C20—C19—C18	117.80 (18)
C5—C6—S1	118.30 (14)	C20—C19—H19	121.1
C8—C7—C12	122.17 (17)	C18—C19—H19	121.1
C8—C7—N1	130.44 (16)	C21—C20—C19	122.93 (17)
C12—C7—N1	107.38 (15)	C21—C20—N2	119.08 (18)
C9—C8—C7	117.49 (18)	C19—C20—N2	117.98 (18)
C9—C8—H8	121.3	C20—C21—C22	118.39 (17)
C7—C8—H8	121.3	C20—C21—H21	120.8
C8—C9—C10	121.45 (18)	C22—C21—H21	120.8
C8—C9—H9	119.3	C21—C22—C17	120.29 (19)
C10—C9—H9	119.3	C21—C22—H22	119.9
C11—C10—C9	120.78 (18)	C17—C22—H22	119.9
C11—C10—H10	119.6	C14—N1—C7	108.58 (13)
C9—C10—H10	119.6	C14—N1—S1	127.66 (12)
C10—C11—C12	119.00 (18)	C7—N1—S1	121.43 (12)
C10—C11—H11	120.5	O3—N2—O4	123.39 (19)
C12—C11—H11	120.5	O3—N2—C20	118.71 (18)
C11—C12—C7	119.05 (16)	O4—N2—C20	117.9 (2)
C11—C12—C13	133.69 (16)	O2—S1—O1	119.98 (9)
C7—C12—C13	107.21 (15)	O2—S1—N1	106.48 (8)
C14—C13—C12	108.61 (15)	O1—S1—N1	106.25 (8)
C14—C13—C16	125.31 (16)	O2—S1—C6	110.14 (9)
C12—C13—C16	125.96 (15)	O1—S1—C6	108.72 (9)
C13—C14—N1	108.19 (15)	N1—S1—C6	104.01 (8)
C13—C14—C15	127.68 (16)		
			
C6—C1—C2—C3	0.4 (5)	C22—C17—C18—C19	0.9 (3)
C1—C2—C3—C4	−1.2 (5)	C16—C17—C18—C19	179.71 (17)
C2—C3—C4—C5	1.2 (4)	C17—C18—C19—C20	1.3 (3)
C3—C4—C5—C6	−0.4 (4)	C18—C19—C20—C21	−2.9 (3)
C2—C1—C6—C5	0.4 (4)	C18—C19—C20—N2	175.93 (17)
C2—C1—C6—S1	−178.8 (2)	C19—C20—C21—C22	2.2 (3)
C4—C5—C6—C1	−0.4 (3)	N2—C20—C21—C22	−176.61 (17)
C4—C5—C6—S1	178.80 (18)	C20—C21—C22—C17	0.1 (3)
C12—C7—C8—C9	1.2 (3)	C18—C17—C22—C21	−1.6 (3)
N1—C7—C8—C9	−179.64 (17)	C16—C17—C22—C21	179.56 (18)
C7—C8—C9—C10	1.2 (3)	C13—C14—N1—C7	1.69 (19)
C8—C9—C10—C11	−2.0 (3)	C15—C14—N1—C7	176.00 (17)
C9—C10—C11—C12	0.5 (3)	C13—C14—N1—S1	164.34 (13)
C10—C11—C12—C7	1.8 (3)	C15—C14—N1—S1	−21.3 (3)
C10—C11—C12—C13	178.71 (19)	C8—C7—N1—C14	179.50 (18)
C8—C7—C12—C11	−2.7 (3)	C12—C7—N1—C14	−1.21 (18)
N1—C7—C12—C11	177.97 (15)	C8—C7—N1—S1	15.6 (3)
C8—C7—C12—C13	179.66 (16)	C12—C7—N1—S1	−165.15 (12)
N1—C7—C12—C13	0.30 (18)	C21—C20—N2—O3	−177.00 (19)
C11—C12—C13—C14	−176.44 (18)	C19—C20—N2—O3	4.1 (3)
C7—C12—C13—C14	0.7 (2)	C21—C20—N2—O4	3.2 (3)
C11—C12—C13—C16	7.5 (3)	C19—C20—N2—O4	−175.7 (2)
C7—C12—C13—C16	−175.30 (17)	C14—N1—S1—O2	23.27 (17)
C12—C13—C14—N1	−1.5 (2)	C7—N1—S1—O2	−176.07 (13)
C16—C13—C14—N1	174.58 (16)	C14—N1—S1—O1	152.25 (15)
C12—C13—C14—C15	−175.52 (17)	C7—N1—S1—O1	−47.09 (15)
C16—C13—C14—C15	0.6 (3)	C14—N1—S1—C6	−93.08 (16)
C14—C13—C16—O5	40.2 (3)	C7—N1—S1—C6	67.58 (14)
C12—C13—C16—O5	−144.4 (2)	C1—C6—S1—O2	144.10 (17)
C14—C13—C16—C17	−138.39 (18)	C5—C6—S1—O2	−35.13 (18)
C12—C13—C16—C17	37.0 (3)	C1—C6—S1—O1	10.8 (2)
O5—C16—C17—C22	36.7 (3)	C5—C6—S1—O1	−168.46 (15)
C13—C16—C17—C22	−144.65 (18)	C1—C6—S1—N1	−102.14 (18)
O5—C16—C17—C18	−142.2 (2)	C5—C6—S1—N1	78.64 (16)
C13—C16—C17—C18	36.5 (3)		

### 1-Benzenesulfonyl-2-methyl-3-(4-nitrobenzoyl)-2,3-dihydro-1H-indole (I) Hydrogen-bond geometry (Å, º)

**Table:**

*D*—H···*A*	*D*—H	H···*A*	*D*···*A*	*D*—H···*A*
C8—H8···O1	0.93	2.40	2.977 (3)	120
C8—H8···O4^i^	0.93	2.60	3.286 (2)	131
C15—H15*A*···O2	0.96	2.03	2.824 (3)	139
C15—H15*A*···S1	0.96	2.84	3.307 (2)	111
C19—H19···O2^ii^	0.93	2.64	3.388 (2)	138

Symmetry codes: (i) −*x*+3/2, *y*+1/2, −*z*+1/2; (ii) *x*−1/2, −*y*+3/2, *z*+1/2.

### 1-Benzenesulfonyl-2-methyl-3-[(thiophen-2-yl)carbonyl]-2,3-dihydro-1H-indole (II) Crystal data

**Table:**

C_20_H_15_NO_3_S_2_	*D*_x_ = 1.408 Mg m^−^^3^
*M**_r_* = 381.45	Cu *K*α radiation, λ = 1.54178 Å
Orthorhombic, *P*2_1_2_1_2_1_	Cell parameters from 9899 reflections
*a* = 8.9300 (2) Å	θ = 4.7–72.4°
*b* = 10.8141 (3) Å	µ = 2.85 mm^−^^1^
*c* = 18.6398 (5) Å	*T* = 293 K
*V* = 1800.04 (8) Å^3^	Block, colourless
*Z* = 4	0.25 × 0.20 × 0.15 mm
*F*(000) = 792	

### 1-Benzenesulfonyl-2-methyl-3-[(thiophen-2-yl)carbonyl]-2,3-dihydro-1H-indole (II) Data collection

**Table:**

Bruker Kappa APEX3 CMOS diffractometer	3538 independent reflections
Radiation source: micro-focus sealed tube	3314 reflections with *I* > 2σ(*I*)
Graphite monochromator	*R*_int_ = 0.043
ω and φ scan	θ_max_ = 72.4°, θ_min_ = 4.7°
Absorption correction: multi-scan (SADABS; Bruker, 2016)	*h* = −10→11
*T*_min_ = 0.599, *T*_max_ = 0.746	*k* = −13→13
25415 measured reflections	*l* = −19→22

### 1-Benzenesulfonyl-2-methyl-3-[(thiophen-2-yl)carbonyl]-2,3-dihydro-1H-indole (II) Refinement

**Table:**

Refinement on *F*^2^	Hydrogen site location: inferred from neighbouring sites
Least-squares matrix: full	H-atom parameters constrained
*R*[*F*^2^ > 2σ(*F*^2^)] = 0.039	*w* = 1/[σ^2^(*F*_o_^2^) + (0.0632*P*)^2^ + 0.446*P*] where *P* = (*F*_o_^2^ + 2*F*_c_^2^)/3
*wR*(*F*^2^) = 0.106	(Δ/σ)_max_ = 0.002
*S* = 1.06	Δρ_max_ = 0.24 e Å^−^^3^
3538 reflections	Δρ_min_ = −0.38 e Å^−^^3^
236 parameters	Absolute structure: Refined as an inversion twin.
0 restraints	Absolute structure parameter: 0.03 (3)

### 1-Benzenesulfonyl-2-methyl-3-[(thiophen-2-yl)carbonyl]-2,3-dihydro-1H-indole (II) Special details

**Table:**

Geometry. All esds (except the esd in the dihedral angle between two l.s. planes) are estimated using the full covariance matrix. The cell esds are taken into account individually in the estimation of esds in distances, angles and torsion angles; correlations between esds in cell parameters are only used when they are defined by crystal symmetry. An approximate (isotropic) treatment of cell esds is used for estimating esds involving l.s. planes.
Refinement. Refined as a 2-component inversion twin.

### 1-Benzenesulfonyl-2-methyl-3-[(thiophen-2-yl)carbonyl]-2,3-dihydro-1H-indole (II) Fractional atomic coordinates and isotropic or equivalent isotropic displacement parameters (Å^2^)

**Table:**

	*x*	*y*	*z*	*U*_iso_*/*U*_eq_	
C1	0.9645 (4)	0.1139 (3)	0.89175 (17)	0.0390 (6)	
C2	0.8811 (4)	0.2195 (3)	0.8753 (2)	0.0500 (8)	
H2	0.8773	0.2508	0.8289	0.060*	
C3	0.8044 (5)	0.2755 (3)	0.9311 (2)	0.0595 (10)	
H3	0.7477	0.3458	0.9218	0.071*	
C4	0.8097 (5)	0.2300 (4)	0.9998 (3)	0.0639 (11)	
H4	0.7571	0.2703	1.0359	0.077*	
C5	0.8917 (5)	0.1255 (3)	1.01619 (19)	0.0537 (8)	
H5	0.8936	0.0946	1.0627	0.064*	
C6	0.9716 (4)	0.0674 (3)	0.96149 (16)	0.0386 (6)	
C7	1.0648 (4)	−0.0418 (3)	0.95980 (16)	0.0386 (6)	
C8	1.1175 (3)	−0.0574 (3)	0.89200 (15)	0.0383 (6)	
C9	1.0953 (4)	−0.1171 (3)	1.02446 (16)	0.0441 (7)	
C10	1.0808 (4)	−0.2510 (3)	1.02034 (18)	0.0450 (7)	
C11	1.0118 (4)	−0.3236 (3)	0.9675 (2)	0.0516 (8)	
H11	0.9682	−0.2936	0.9257	0.062*	
C12	1.0188 (6)	−0.4502 (4)	0.9878 (3)	0.0834 (15)	
H12	0.9795	−0.5136	0.9598	0.100*	
C13	1.0869 (6)	−0.4699 (4)	1.0506 (4)	0.0863 (17)	
H13	1.1005	−0.5481	1.0703	0.104*	
C14	1.2277 (4)	−0.1503 (4)	0.86485 (19)	0.0513 (8)	
H14A	1.2427	−0.1383	0.8143	0.077*	
H14B	1.3212	−0.1401	0.8895	0.077*	
H14C	1.1899	−0.2322	0.8732	0.077*	
C15	1.2007 (5)	0.3125 (4)	0.7758 (2)	0.0600 (10)	
H15	1.1014	0.3357	0.7692	0.072*	
C16	1.3106 (6)	0.4008 (4)	0.7866 (2)	0.0718 (12)	
H16	1.2848	0.4841	0.7873	0.086*	
C17	1.4559 (6)	0.3669 (5)	0.7961 (2)	0.0726 (14)	
H17	1.5288	0.4270	0.8033	0.087*	
C18	1.4952 (5)	0.2442 (6)	0.7951 (3)	0.0783 (14)	
H18	1.5949	0.2215	0.8011	0.094*	
C19	1.3879 (5)	0.1545 (4)	0.7853 (2)	0.0647 (10)	
H19	1.4142	0.0713	0.7855	0.078*	
C20	1.2414 (4)	0.1891 (3)	0.77510 (15)	0.0438 (7)	
N1	1.0537 (3)	0.0363 (3)	0.84821 (14)	0.0412 (6)	
O1	0.9748 (3)	0.1284 (3)	0.73280 (14)	0.0634 (7)	
O2	1.1698 (3)	−0.0325 (3)	0.73361 (13)	0.0601 (7)	
O3	1.1254 (4)	−0.0666 (3)	1.08138 (13)	0.0700 (8)	
S1	1.10413 (10)	0.07464 (8)	0.76447 (4)	0.0452 (2)	
S2	1.14511 (13)	−0.33973 (11)	1.09014 (6)	0.0702 (3)	

### 1-Benzenesulfonyl-2-methyl-3-[(thiophen-2-yl)carbonyl]-2,3-dihydro-1H-indole (II) Atomic displacement parameters (Å^2^)

**Table:**

	*U*^11^	*U*^22^	*U*^33^	*U*^12^	*U*^13^	*U*^23^
C1	0.0334 (14)	0.0344 (14)	0.0491 (16)	−0.0031 (12)	−0.0043 (12)	0.0011 (12)
C2	0.0415 (18)	0.0382 (15)	0.070 (2)	−0.0011 (15)	−0.0045 (16)	0.0110 (15)
C3	0.056 (2)	0.0360 (16)	0.087 (3)	0.0061 (16)	−0.0090 (19)	−0.0058 (18)
C4	0.060 (2)	0.054 (2)	0.077 (3)	0.0106 (19)	0.000 (2)	−0.0211 (19)
C5	0.061 (2)	0.0490 (17)	0.0506 (17)	0.0023 (18)	0.0018 (17)	−0.0108 (14)
C6	0.0402 (15)	0.0315 (13)	0.0440 (15)	−0.0060 (13)	−0.0057 (12)	−0.0036 (12)
C7	0.0416 (16)	0.0340 (14)	0.0401 (14)	−0.0023 (12)	−0.0068 (12)	−0.0029 (11)
C8	0.0354 (15)	0.0352 (14)	0.0445 (15)	−0.0016 (13)	−0.0072 (11)	−0.0008 (11)
C9	0.0473 (17)	0.0420 (15)	0.0428 (15)	−0.0018 (14)	−0.0068 (13)	0.0017 (13)
C10	0.0398 (17)	0.0424 (16)	0.0527 (18)	0.0019 (14)	0.0013 (14)	0.0067 (14)
C11	0.052 (2)	0.0382 (16)	0.065 (2)	−0.0033 (16)	−0.0012 (17)	−0.0038 (15)
C12	0.079 (3)	0.043 (2)	0.128 (5)	−0.013 (2)	0.021 (3)	−0.013 (3)
C13	0.082 (3)	0.050 (2)	0.127 (4)	0.020 (2)	0.034 (3)	0.038 (3)
C14	0.0507 (19)	0.053 (2)	0.0501 (17)	0.0134 (17)	−0.0032 (15)	−0.0014 (15)
C15	0.059 (2)	0.053 (2)	0.068 (2)	−0.0083 (17)	0.0067 (18)	0.0122 (18)
C16	0.089 (3)	0.057 (2)	0.070 (2)	−0.022 (2)	0.007 (2)	0.003 (2)
C17	0.081 (3)	0.084 (3)	0.052 (2)	−0.045 (3)	0.0029 (19)	−0.001 (2)
C18	0.052 (3)	0.106 (4)	0.076 (3)	−0.020 (3)	−0.004 (2)	0.000 (3)
C19	0.049 (2)	0.068 (2)	0.078 (2)	−0.002 (2)	0.0010 (19)	−0.005 (2)
C20	0.0480 (17)	0.0508 (18)	0.0327 (14)	−0.0072 (15)	−0.0005 (12)	−0.0012 (13)
N1	0.0394 (14)	0.0424 (14)	0.0418 (13)	0.0013 (12)	−0.0022 (10)	0.0064 (11)
O1	0.0593 (15)	0.0759 (17)	0.0551 (14)	−0.0082 (14)	−0.0233 (13)	0.0150 (13)
O2	0.0798 (18)	0.0567 (14)	0.0438 (12)	−0.0059 (13)	0.0013 (13)	−0.0136 (11)
O3	0.109 (2)	0.0565 (14)	0.0448 (13)	−0.0037 (17)	−0.0234 (14)	−0.0046 (11)
S1	0.0493 (4)	0.0498 (4)	0.0366 (3)	−0.0066 (3)	−0.0083 (3)	0.0014 (3)
S2	0.0630 (6)	0.0727 (7)	0.0750 (6)	0.0103 (5)	0.0035 (5)	0.0337 (5)

### 1-Benzenesulfonyl-2-methyl-3-[(thiophen-2-yl)carbonyl]-2,3-dihydro-1H-indole (II) Geometric parameters (Å, º)

**Table:**

C1—C6	1.395 (4)	C12—C13	1.337 (8)
C1—C2	1.397 (4)	C12—H12	0.9300
C1—N1	1.413 (4)	C13—S2	1.672 (6)
C2—C3	1.385 (6)	C13—H13	0.9300
C2—H2	0.9300	C14—H14A	0.9600
C3—C4	1.372 (6)	C14—H14B	0.9600
C3—H3	0.9300	C14—H14C	0.9600
C4—C5	1.381 (6)	C15—C20	1.383 (5)
C4—H4	0.9300	C15—C16	1.384 (6)
C5—C6	1.394 (5)	C15—H15	0.9300
C5—H5	0.9300	C16—C17	1.360 (8)
C6—C7	1.446 (4)	C16—H16	0.9300
C7—C8	1.359 (4)	C17—C18	1.373 (8)
C7—C9	1.480 (4)	C17—H17	0.9300
C8—N1	1.421 (4)	C18—C19	1.376 (6)
C8—C14	1.494 (5)	C18—H18	0.9300
C9—O3	1.224 (4)	C19—C20	1.374 (6)
C9—C10	1.455 (5)	C19—H19	0.9300
C10—C11	1.402 (5)	C20—S1	1.753 (3)
C10—S2	1.716 (3)	N1—S1	1.677 (3)
C11—C12	1.421 (6)	O1—S1	1.421 (3)
C11—H11	0.9300	O2—S1	1.421 (3)
			
C6—C1—C2	121.5 (3)	C12—C13—H13	123.4
C6—C1—N1	107.2 (3)	S2—C13—H13	123.4
C2—C1—N1	131.3 (3)	C8—C14—H14A	109.5
C3—C2—C1	117.2 (3)	C8—C14—H14B	109.5
C3—C2—H2	121.4	H14A—C14—H14B	109.5
C1—C2—H2	121.4	C8—C14—H14C	109.5
C4—C3—C2	121.8 (3)	H14A—C14—H14C	109.5
C4—C3—H3	119.1	H14B—C14—H14C	109.5
C2—C3—H3	119.1	C20—C15—C16	118.7 (4)
C3—C4—C5	121.2 (4)	C20—C15—H15	120.6
C3—C4—H4	119.4	C16—C15—H15	120.6
C5—C4—H4	119.4	C17—C16—C15	120.6 (5)
C4—C5—C6	118.6 (4)	C17—C16—H16	119.7
C4—C5—H5	120.7	C15—C16—H16	119.7
C6—C5—H5	120.7	C16—C17—C18	120.2 (4)
C5—C6—C1	119.7 (3)	C16—C17—H17	119.9
C5—C6—C7	132.8 (3)	C18—C17—H17	119.9
C1—C6—C7	107.5 (3)	C17—C18—C19	120.4 (5)
C8—C7—C6	108.7 (3)	C17—C18—H18	119.8
C8—C7—C9	128.7 (3)	C19—C18—H18	119.8
C6—C7—C9	122.5 (3)	C20—C19—C18	119.3 (4)
C7—C8—N1	107.9 (3)	C20—C19—H19	120.4
C7—C8—C14	128.8 (3)	C18—C19—H19	120.4
N1—C8—C14	123.3 (3)	C19—C20—C15	120.8 (4)
O3—C9—C10	120.6 (3)	C19—C20—S1	119.2 (3)
O3—C9—C7	120.1 (3)	C15—C20—S1	119.9 (3)
C10—C9—C7	119.2 (3)	C1—N1—C8	108.7 (2)
C11—C10—C9	129.3 (3)	C1—N1—S1	122.6 (2)
C11—C10—S2	111.5 (3)	C8—N1—S1	127.1 (2)
C9—C10—S2	119.1 (3)	O2—S1—O1	120.08 (18)
C10—C11—C12	109.5 (4)	O2—S1—N1	106.61 (15)
C10—C11—H11	125.2	O1—S1—N1	105.64 (16)
C12—C11—H11	125.2	O2—S1—C20	109.46 (17)
C13—C12—C11	114.0 (5)	O1—S1—C20	109.02 (17)
C13—C12—H12	123.0	N1—S1—C20	104.91 (14)
C11—C12—H12	123.0	C13—S2—C10	91.9 (2)
C12—C13—S2	113.2 (3)		
			
C6—C1—C2—C3	−0.7 (5)	C20—C15—C16—C17	0.2 (7)
N1—C1—C2—C3	180.0 (3)	C15—C16—C17—C18	0.0 (7)
C1—C2—C3—C4	0.2 (6)	C16—C17—C18—C19	−0.7 (7)
C2—C3—C4—C5	−0.3 (6)	C17—C18—C19—C20	1.2 (7)
C3—C4—C5—C6	0.9 (6)	C18—C19—C20—C15	−1.1 (6)
C4—C5—C6—C1	−1.4 (5)	C18—C19—C20—S1	−178.7 (3)
C4—C5—C6—C7	−179.0 (3)	C16—C15—C20—C19	0.4 (6)
C2—C1—C6—C5	1.3 (5)	C16—C15—C20—S1	178.0 (3)
N1—C1—C6—C5	−179.2 (3)	C6—C1—N1—C8	−0.8 (3)
C2—C1—C6—C7	179.4 (3)	C2—C1—N1—C8	178.6 (3)
N1—C1—C6—C7	−1.0 (3)	C6—C1—N1—S1	−167.3 (2)
C5—C6—C7—C8	−179.6 (4)	C2—C1—N1—S1	12.1 (5)
C1—C6—C7—C8	2.6 (3)	C7—C8—N1—C1	2.5 (3)
C5—C6—C7—C9	−1.1 (6)	C14—C8—N1—C1	−175.2 (3)
C1—C6—C7—C9	−178.9 (3)	C7—C8—N1—S1	168.2 (2)
C6—C7—C8—N1	−3.1 (3)	C14—C8—N1—S1	−9.5 (4)
C9—C7—C8—N1	178.5 (3)	C1—N1—S1—O2	−170.3 (3)
C6—C7—C8—C14	174.4 (3)	C8—N1—S1—O2	25.8 (3)
C9—C7—C8—C14	−4.0 (6)	C1—N1—S1—O1	−41.5 (3)
C8—C7—C9—O3	134.4 (4)	C8—N1—S1—O1	154.7 (3)
C6—C7—C9—O3	−43.8 (5)	C1—N1—S1—C20	73.7 (3)
C8—C7—C9—C10	−49.0 (5)	C8—N1—S1—C20	−90.2 (3)
C6—C7—C9—C10	132.8 (3)	C19—C20—S1—O2	−28.5 (3)
O3—C9—C10—C11	162.0 (4)	C15—C20—S1—O2	153.8 (3)
C7—C9—C10—C11	−14.7 (6)	C19—C20—S1—O1	−161.7 (3)
O3—C9—C10—S2	−14.0 (5)	C15—C20—S1—O1	20.7 (3)
C7—C9—C10—S2	169.4 (3)	C19—C20—S1—N1	85.5 (3)
C9—C10—C11—C12	−177.2 (4)	C15—C20—S1—N1	−92.1 (3)
S2—C10—C11—C12	−1.0 (4)	C12—C13—S2—C10	−1.1 (4)
C10—C11—C12—C13	0.2 (6)	C11—C10—S2—C13	1.2 (3)
C11—C12—C13—S2	0.7 (6)	C9—C10—S2—C13	177.8 (3)

### 1-Benzenesulfonyl-2-methyl-3-[(thiophen-2-yl)carbonyl]-2,3-dihydro-1H-indole (II) Hydrogen-bond geometry (Å, º)

**Table:**

*D*—H···*A*	*D*—H	H···*A*	*D*···*A*	*D*—H···*A*
C2—H2···O1	0.93	2.39	2.954 (5)	119
C2—H2···O2^i^	0.93	2.65	3.394 (4)	138
C14—H14*A*···O2	0.96	2.00	2.806 (4)	140
C14—H14*A*···S1	0.96	2.77	3.261 (4)	112
C14—H14*B*···S2^ii^	0.96	2.93	3.822 (4)	156

Symmetry codes: (i) −*x*+2, *y*+1/2, −*z*+3/2; (ii) *x*+1/2, −*y*−1/2, −*z*+2.
